# Efficacy and Safety of High-Specific-Activity ^131^I-MIBG Therapy in Patients with Advanced Pheochromocytoma or Paraganglioma

**DOI:** 10.2967/jnumed.118.217463

**Published:** 2019-05

**Authors:** Daniel A. Pryma, Bennett B. Chin, Richard B. Noto, Joseph S. Dillon, Stephanie Perkins, Lilja Solnes, Lale Kostakoglu, Aldo N. Serafini, Miguel H. Pampaloni, Jessica Jensen, Thomas Armor, Tess Lin, Theresa White, Nancy Stambler, Stuart Apfel, Vincent A. DiPippo, Syed Mahmood, Vivien Wong, Camilo Jimenez

**Affiliations:** 1 Department of Radiology, University of Pennsylvania School of Medicine, Philadelphia, Pennsylvania; 2 Department of Radiology–Nuclear Medicine, University of Colorado Anschutz Medical Campus, Denver, Colorado; 3 Division of Nuclear Medicine, Rhode Island Hospital/Warren Alpert Medical School of Brown University, Providence, Rhode Island; 4 Department of Internal Medicine, University of Iowa Carver College of Medicine, Iowa City, Iowa; 5 Department of Radiation Oncology, Washington University School of Medicine, St. Louis, Missouri; 6 Department of Radiology and Radiological Science, Johns Hopkins Medicine, Baltimore, Maryland; 7 Department of Nuclear Medicine, Icahn School of Medicine at Mount Sinai, New York, New York; 8 Division of Nuclear Medicine, University of Miami School of Medicine, Miami, Florida; 9 Department of Radiology and Biomedical Imaging, University of California at San Francisco School of Medicine, San Francisco, California; 10 Department of Research and Development, Progenics Pharmaceuticals, Inc., New York, New York; and; 11 Department of Endocrine Neoplasia and Hormonal Disorders, University of Texas M.D. Anderson Cancer Center, Houston, Texas

**Keywords:** high-specific-activity ^131^I-MIBG, neuroendocrine tumors, paraganglioma, pheochromocytoma, rare, ultra-orphan disease

## Abstract

Patients with metastatic or unresectable (advanced) pheochromocytoma and paraganglioma (PPGL) have poor prognoses and few treatment options. This multicenter, phase 2 trial evaluated the efficacy and safety of high-specific-activity ^131^I-meta-iodobenzylguanidine (HSA ^131^I-MIBG) in patients with advanced PPGL. **Methods:** In this open-label, single-arm study, 81 PPGL patients were screened for enrollment, and 74 received a treatment-planning dose of HSA ^131^I-MIBG. Of these patients, 68 received at least 1 therapeutic dose (∼18.5 GBq) of HSA ^131^I-MIBG intravenously. The primary endpoint was the proportion of patients with at least a 50% reduction in baseline antihypertensive medication use lasting at least 6 mo. Secondary endpoints included objective tumor response as assessed by Response Evaluation Criteria in Solid Tumors version 1.0, biochemical tumor marker response, overall survival, and safety. **Results:** Of the 68 patients who received at least 1 therapeutic dose of HSA ^131^I-MIBG, 17 (25%; 95% confidence interval, 16%–37%) had a durable reduction in baseline antihypertensive medication use. Among 64 patients with evaluable disease, 59 (92%) had a partial response or stable disease as the best objective response within 12 mo. Decreases in elevated (≥1.5 times the upper limit of normal at baseline) serum chromogranin levels were observed, with confirmed complete and partial responses 12 mo after treatment in 19 of 28 patients (68%). The median overall survival was 36.7 mo (95% confidence interval, 29.9–49.1 mo). The most common treatment-emergent adverse events were nausea, myelosuppression, and fatigue. No patients had drug-related acute hypertensive events during or after the administration of HSA ^131^I-MIBG. **Conclusion:** HSA ^131^I-MIBG offers multiple benefits, including sustained blood pressure control and tumor response in PPGL patients.

Pheochromocytomas and paragangliomas (PPGLs) are rare neuroendocrine tumors with an incidence of between 2 and 8 cases per million per year (1–3). Most PPGLs hypersecrete catecholamines, which can cause hypertension, arrhythmias, and headaches, leading to considerable morbidity and mortality (1–3). The standard first-line intervention for localized PPGL is surgery, which can be curative; however, 10%–35% of PPGLs are locally invasive or metastatic and not amenable to curative surgery (1–4). Estimates of 5-y survival rates vary (12%–60%) with the location of metastatic lesions (2,5); poorer prognoses have been reported for patients who have liver or lung metastases or present with predominant bone metastases (6,7). Shorter survival has been correlated with synchronous metastases, large primary tumor size, dependence on primary tumor location, germline mutations of the succinate dehydrogenase subunit B gene, and unresectable primary tumor (4,8).

Lacking therapies approved by the U.S. Food and Drug Administration for the treatment of their disease, patients with advanced PPGL have few treatment options (9). Current treatment paradigms include the use of cytotoxic chemotherapy with cyclophosphamide, vincristine, and dacarbazine and conventional ^131^I-meta-iodobenzylguanidine (^131^I-MIBG) (9–14). A metaanalysis of the use of cyclophosphamide, vincristine, and dacarbazine chemotherapy for malignant PPGL showed that complete responses (CRs) and partial responses (PRs) as assessed by reductions in tumor volume were achieved in 4% and 37% of patients, respectively; however, the designs and response criteria of the studies included in the analysis were highly variable (13). MIBG, a guanethidine derivative, is a substrate for norepinephrine reuptake transporters, which are highly expressed on the cell surface of neuroendocrine tumors such as PPGL (14). In conventional preparations, more than 99% of the MIBG molecules are not labeled with radioactive iodine (^131^I), resulting in products with very low specific activity (3.3 mCi/mg; 123.3 MBq/mg) (15). This in turn results in the administration of large doses of unlabeled MIBG, which compete for norepinephrine reuptake transporter binding sites and disrupt the norepinephrine-reuptake mechanism (12,14). The resulting increase in circulating catecholamines can lead to life-threatening side effects such as acute hypertensive crisis during or shortly after drug administration (12,14). Therefore, a formulation of radiolabeled MIBG that contains a significantly smaller amount of unlabeled drug could reduce this side effect.

High-specific-activity (HSA, ∼2,500 mCi/mg; 92,500 MBq/mg) ^131^I-MIBG is a targeted therapeutic that consists almost entirely of ^131^I-labeled MIBG (14,16–19). HSA ^131^I-MIBG showed antitumor activity in patients with metastatic or unresectable PPGL in a phase 1 trial and has received breakthrough therapy designation for the treatment of patients with this ultra-orphan disease (19–21). In this first-of-its-kind phase 2 trial, we evaluated the efficacy and safety of HSA ^131^I-MIBG in patients with metastatic or unresectable (advanced) PPGL. The successful management of advanced PPGL is challenging and requires a multidisciplinary approach to control endocrine activity, decrease tumor burden, and alleviate debilitating symptoms. Advanced PPGL is rare and has a long natural history; therefore, a randomized trial with a survival endpoint would be essentially impossible. Alternatively, a small improvement in blood pressure (BP) control may reduce cardiovascular complications and prolong survival in patients with metastatic PPGL (9,10,22); thus, control of tumor-associated hypertension is critical in the management of patients with metastatic PPGL. Approximately two thirds of patients with PPGLs present with hypertension secondary to an excessive secretion of catecholamines (noradrenaline–adrenaline) (23). Hypertension may predispose to myocardial infarction, angina, diastolic dysfunction, stroke, retinopathy, and renal failure. Furthermore, the excessive secretion of catecholamines may cause intestinal ischemia, arrhythmia, and catecholamine cardiomyopathy (23). The secretion of catecholamines is proportional to the tumor burden. Most patients with metastatic PPGLs have a large tumor burden; subsequently, hypertension can be difficult to control. Because hypertension is a significant cause of morbidity for these patients and can be objectively measured, as requested by the Food and Drug Administration, a stringently defined reduction in antihypertensive use was chosen as the primary endpoint of this study.

## MATERIALS AND METHODS

The study protocol and all amendments were approved by each study center’s Institutional Review Board. The trial was performed in accordance with the Declaration of Helsinki, the International Conference on Harmonisation Good Clinical Practice guidelines, and all applicable regulations. An independent data-monitoring committee was established to safeguard the integrity of the study and assess the safety and efficacy of the interventions. Patients (or, for patients younger than 18 y, legal guardians) provided written informed consent for study participation.

### Trial Design

This multicenter, open-label, single-arm trial, which was conducted under a Special Protocol Assessment agreement with the Food and Drug Administration, consisted of a 1-y efficacy phase followed by a 4-y long-term follow-up phase (ClinicalTrials.gov: NCT00874614). As per the Special Protocol Assessment agreement, the primary study endpoint was a clinical surrogate endpoint related to a significant symptom seen in patients. The primary endpoint was defined as at least a 50% reduction in baseline antihypertensive medication lasting at least 6 mo, beginning in the 12-mo efficacy phase, without the introduction of new long-term (>14 d) antihypertensive medication or increases in the doses of the baseline regimen. Secondary objectives included radiographic tumor response, biochemical tumor marker response, overall survival (OS), and safety.

### Patients

Patients were screened for enrollment at 10 centers in the United States between June 2009 and February 2016. Eligible patients were at least 12 y old, had a documented PPGL diagnosis confirmed by histology or by a physician using other supportive data (e.g., abnormal results on a ^123/131^I-MIBG diagnostic study or elevated tumor marker levels), were ineligible for curative surgery, progressed on prior therapy for PPGL or were not candidates for chemotherapy or other therapy, had hypertension secondary to a catecholamine excess, and had been on a stable antihypertensive medication regimen (defined as no addition or deletion of antihypertensive medication and no change in total daily dose or route of administration for currently used antihypertensive medication) for at least 30 d before the first therapeutic dose of HSA ^131^I-MIBG. Eligibility criteria required patients’ tumors to have definitive iobenguane avidity; at least 1 metastatic and not resectable tumor site identified by CT, MRI, or iobenguane ^131^I scanning; a Karnofsky performance status of least 60; and absence of active central nervous system lesions.

Patients were excluded if they had received any previous systemic radiotherapy resulting in bone marrow toxicity within 3 mo of study entry; had a malignancy other than PPGL that required treatment during the present trial; had received chemotherapy within 30 d of the first therapeutic dose of HSA ^131^I-MIBG; or had a platelet count of less than 80,000/μL, an absolute neutrophil count of less than 1,200 cells/μL, or creatinine clearance of less than 30 mL/min.

One patient received HSA ^131^I-MIBG in a prior clinical trial and received therapeutic doses in this study (20).

### Treatment

Eligible patients received an intravenous treatment-planning dose of HSA ^131^I-MIBG (∼0.185 GBq [5 mCi]) and then underwent serial whole-body scans for assessing MIBG avidity and biodistribution and for performing dosimetry calculations to determine normal organs’ absorbed radiation doses as described previously (24,25). Calculations of estimated mean absorbed radiation dose were performed using OLINDA/EXM software (version 1.1; Vanderbilt University) and are summarized in Supplemental Table 1 (supplemental materials are available at http://jnm.snmjournals.org). Patients with MIBG-avid tumors (Supplemental Fig. 1, panels 1 and 2) after the treatment-planning dose received 1 or 2 therapeutic doses of HSA ^131^I-MIBG (∼18.5 GBq [500 mCi] or, for patients ≤ 62.5 kg, 0.296 GBq/kg [8 mCi/kg]), administered intravenously (infusion over 30 min in an inpatient setting) approximately 90 d apart. The dosing regimen was based on the previous dose-ranging study of HSA ^131^I-MIBG by Noto et al. (20). To ensure that critical organs’ absorbed radiation doses would not exceed established toxicity limits after 2 therapeutic doses, we performed individualized dose reduction as described previously (26). Patients underwent electrocardiography before, during, and after each therapeutic dose. Whole-body scans were acquired within 7 d after each therapeutic dose to assess the biodistribution and tumor uptake of HSA ^131^I-MIBG. Patients whose hematologic values returned to baseline levels or were within the reference range within 24 wk after the first therapeutic dose were eligible for the second therapeutic dose.

### Efficacy Assessment

At each patient visit, a current list of antihypertensive medications and their doses and a compliance history were obtained. BP and heart rate were measured every time antihypertensive therapy was changed. The professional staff (site staff or visiting nurses) measured BP and heart rate at screening or baseline, twice weekly in the first 6 wk after each therapeutic dose, weekly through week 24 (at weeks when not collected twice weekly), and monthly at months 7 through 12. If the second therapeutic dose was delayed, the twice-weekly or weekly blood measurements would occur past week 24. The twice-weekly measurements occurred for at least 6 wk after the second therapeutic dose, and the weekly measurements continued until at least 12 wk past the second therapeutic dose.

A reduction in antihypertensive medication was considered if the patient’s systolic and diastolic BP values were less than 140 mm Hg and less than 90 mm Hg, respectively, as measured with a manual mercury sphygmomanometer. Between visits, BP measurements were recorded at the same time on different days at least once or twice per week to facilitate titration of the antihypertensive regimen. If the patient’s BP remained within a normotensive range (i.e., systolic BP < 140 mm Hg and diastolic BP < 90 mm Hg), further reduction or discontinuation of antihypertensive medication was considered. Concomitant antihypertensive medications (by class) used by at least 10% of patients included α-adrenoreceptor antagonists (52.7%), selective β-blocking agents (45.9%), selective other peripheral vasodilators (35.1%), angiotensin-converting enzyme inhibitors (21.6%), dihydropyridine derivatives (21.6%), α- and β-blocking agents (14.9%), and nonselective β-blocking agents (13.5%).

### Tumor Response, Biochemical Tumor Marker Response, and Safety Assessments

For the tumor response evaluation, each patient underwent baseline CT or MRI of the chest, abdomen, and pelvis at study entry and underwent follow-up imaging every 3 mo during the 12-mo efficacy phase. Three anterior and posterior whole-body scans were performed for the assessment of dosimetry, biodistribution, and tumor uptake before qualifying to receive HSA ^131^I-MIBG therapy. Image 1 was started within an hour of the end of the dosimetry dose of HSA ^131^I-MIBG, image 2 was performed 1–2 d after dosimetry dosing, and the last image was performed 2–5 d after the dosimetry dose. Subjects had 1 additional scan within 7 d after each of the 2 therapeutic doses of HSA ^131^I-MIBG to further assess biodistribution. Radiographic response according to RECIST version 1.0 was assessed by 2 independent central reviewers and 1 adjudicator (27).

The objective response rate was defined as the percentage of patients who had a response according to RECIST version 1.0. Patients (including patients who had only bone metastases) for whom no postbaseline CT or MRI scans or central response data were available were excluded from the analysis. PRs or CRs on CT or MRI were confirmed by follow-up imaging.

For the biochemical tumor marker response assessment, levels of PPGL-associated markers, including plasma and urine catecholamines and their metabolites and serum chromogranin A, were measured at baseline and throughout the study by a central laboratory. Briefly, samples were collected and analyzed at study entry, every 2 wk after the first therapeutic dose of HSA ^131^I-MIBG (weeks 2–24), and once each month after the second therapeutic dose of the drug (months 7–12). Biomarker overall response was defined as a best confirmed response of either CR or PR at any time for any of the biomarkers whose levels were at least 1.5 times the upper limit of normal at baseline. Biochemical CRs and PRs (normalization of or ≥50% decrease in marker levels, respectively) were confirmed at next assessments.

For the safety assessment, we recorded all adverse events (AEs; graded according to the National Cancer Institute Common Terminology Criteria for Adverse Events version 3.0) and laboratory test abnormalities (hematology, clinical chemistry, and urinalysis) up to 16 wk after the last therapeutic dose of HSA ^131^I-MIBG. As per the study protocol, a serious AE (SAE) was defined as any untoward medical occurrence that resulted in death, was life-threatening, required inpatient hospitalization or prolongation of existing hospitalization, resulted in persistent or significant disability or incapacity, or resulted in a congenital anomaly or birth defect. Secondary malignancies and long-term side effects were captured in the long-term follow-up period.

### Statistical Analysis

The planned sample size for this study was 58 subjects. The alternative hypothesis was a primary endpoint response proportion of 0.25, against the null hypothesis of 0.10 at a 1-sided significance level of α = 0.025 and power of 0.90 (90%). This endpoint was considered achieved if the lower bound of the 2-sided 95% confidence interval (CI) exceeded 0.10 (10%). Patients who received at least 1 therapeutic dose of HSA ^131^I-MIBG were included in the primary analysis. No imputation for missing values, other than partial dates, was performed. Descriptive statistics are presented. Continuous variables are presented as means, SDs, medians, ranges, and sample sizes. Categoric variables are presented as numbers of patients and respective percentages. OS duration, calculated from the date of first therapeutic dose to death, or censored at the last date the patient was known to be alive, was estimated using the Kaplan–Meier product-limit method; 1- through 5-y OS rates are presented as medians and respective 2-sided 95% CIs. All statistical analyses, data listings, tables, and figures (excluding dosimetry analyses) were produced using SAS software (version 9.4; SAS Institute).

## RESULTS

### Patients and Treatment

Of 81 PPGL patients who were screened for enrollment, 74 (safety population) received a treatment-planning dose of HSA ^131^I-MIBG. These patients’ demographic and clinical characteristics are given in Table 1. The median age was 55 y (range, 16–76 y); 55% were men and 45% were women.

**TABLE 1:** Demographics and Baseline Characteristics of 74 Patients with Advanced PPGL Who Received Any Dose of HSA ^131^I-MIBG

Characteristic	Data
Sex	
Male	41 (55)
Female	33 (45)
Age (y)	
Mean ± SD	51.1 ± 13.77
Median	54.5 (range, 16–76)
<18	1 (1)
18–30	7 (10)
31–64	54 (73)
>64	12 (16)
Primary diagnosis	
Pheochromocytoma	53 (72)
Paraganglioma	21 (28)
Prior treatments	
Included surgery	66 (89)
Included conventional ^131^I-MIBG therapy or HSA ^131^I-MIBG	22 (30)
Included chemotherapy with CVD or others	28 (38)
No. of prior treatment modalities	
1	20 (27)
2	26 (35)
3	20 (27)
4	6 (8)
None documented	2 (3)
Location of metastases*	
Lymph nodes	40 (63)
Bone	39 (61)
Lung	22 (34)
Liver	17 (27)
Lung or liver	32 (50)
Bone and lung or liver	20 (31)
Others^†^	24 (38)

* Data provided for 64 patients with evaluable target lesions at baseline.

† Abdominal cavity, adrenal gland, kidney, neck soft tissue, pancreas, pelvic soft tissue, pericardial effusion, peritoneal cavity, peritoneum, pleural cavity, retroperitoneum, or small bowel.

CVD = cyclophosphamide, vincristine, and dacarbazine.

Qualitative data are expressed as numbers followed by percentages in parentheses; continuous data are expressed as mean ± SD.

**FIGURE 1.**
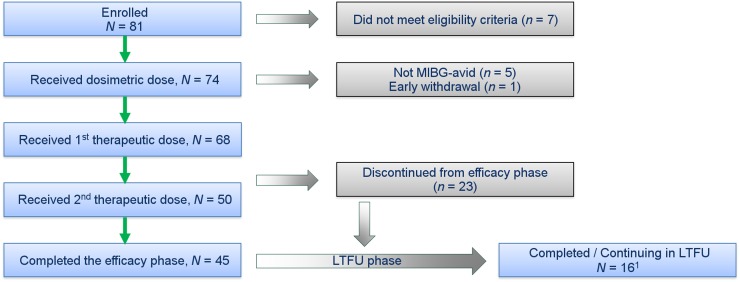
Flowchart of patient disposition. LTFU = long-term follow-up. ^1^Analysis cutoff: December 11, 2017; 81 patients were enrolled in study, and 74 patients received dosimetric dose; of which 68 received 1 therapeutic dose and 50 received 2 therapeutic doses.

Of the 74 patients who received a treatment-planning dose of HSA ^131^I-MIBG, 68 (49 [72%] with pheochromocytoma and 19 [28%] with paraganglioma) had MIBG-avid tumors and received at least 1 therapeutic dose; 50 of these patients (74%) received 2 therapeutic doses. The median cumulative therapeutic HSA ^131^I-MIBG dose was 35.7 GBq (range, 3.8–40.5 GBq; or 965 mCi [range, 102–1,096 mCi]). The median time between doses was 3.45 mo, with a range of 2.7–7.6 mo. One patient received a low dose (3.8 GBq [102 mCi]) because of the patient’s high tumor burden identified at the treatment-planning step, which necessitated a therapeutic dose reduction from the weight-based dose (16.4 GBq [442 mCi]) to what was estimated to be safe to the patient’s kidneys, lungs, and liver (26). Of the 45 patients who completed the 12-mo efficacy phase, 43 entered the long-term follow-up phase. Of the 23 patients who did not complete the efficacy phase, 11 discontinued because of AEs, 6 because of progressive disease, 4 because they received another anticancer therapy, 1 because of withdrawal of consent, and 1 because of loss to follow-up. At the end of 2017, 16 patients remained in the long-term follow-up phase (Fig. 1).

The duration from the date of initial diagnosis until the time of first study treatment was highly variable (1.7 mo to 42.6 y, with a median of 7.3 y for the 68 patients who received therapeutic doses) related to the natural history of PPGL.

### Reduction in Antihypertensive Medication

Of the 68 patients who received at least 1 therapeutic dose of HSA ^131^I-MIBG, 17 (25%; 95% CI, 16%–37%) had an at least 50% reduction in baseline antihypertensive medication lasting at least 6 mo, including 16 of the 50 patients (32%) who received 2 therapeutic doses and 1 of the 18 patients (6%) who received 1 therapeutic dose (Table 2). For these responders, the median duration of reduced baseline antihypertensive medication was 13 mo (range, 8–60 mo). Overall, 33 of the 68 patients (49%) had an at least 50% reduction in antihypertensive medication of any duration. Median duration was 8.0 mo (range, 0.1–60.2 mo). The response durations for each patient are shown in Supplemental Figure 2. The trial achieved the prespecified primary endpoint.

**TABLE 2:** Antihypertensive Medication Reductions and Objective Tumor Responses Among 68 Patients with Advanced PPGL Who Received at Least 1 Dose of HSA ^131^I-MIBG

Parameter	One therapeutic dose (*n* = 18)	Two therapeutic doses (*n* = 50)	At least 1 therapeutic dose (*n* = 68)
Reduction of all antihypertensive medications by ≥50% for ≥6 mo (*n*)			
Yes	1 (6%) (95% CI, 0–28)	16 (32%) (95% CI, 21–46)	17 (25%) (95% CI, 16–37)
No	17 (94%)	34 (68%)	51 (75%)
Best confirmed overall tumor response by RECIST 1.0 (*n*)			
Evaluated patients	14	50	64
CR	0	0	0
PR	0	15 (30%)	15 (23%)
Stable disease	10 (71%)	34 (68%)	44 (69%)
Progressive disease	2 (14%)	1 (2%)	3 (5%)
No assessment	2 (14%)	0	2 (3%)

### Objective Tumor Response

Objective tumor responses among patients who received at least 1 therapeutic dose of HSA ^131^I-MIBG are presented in Table 2. Of the 68 patients who received at least 1 therapeutic dose of HSA ^131^I-MIBG, 4 did not have evaluable target lesions at baseline. Overall, of the 64 patients with evaluable disease, 59 (92%) had PRs (15 patients) or stable disease (44 patients); no patients had a CR. Of the 50 patients who received 2 therapeutic doses and had evaluable disease, 15 (30%) had PRs. A representative image of a PR is provided in Supplemental Figure 1, panel 3. Among the 17 patients who did and the 47 patients who did not have a reduction in antihypertensive medication, 7 (41%) and 8 (17%), respectively, had PRs.

**FIGURE 2.**
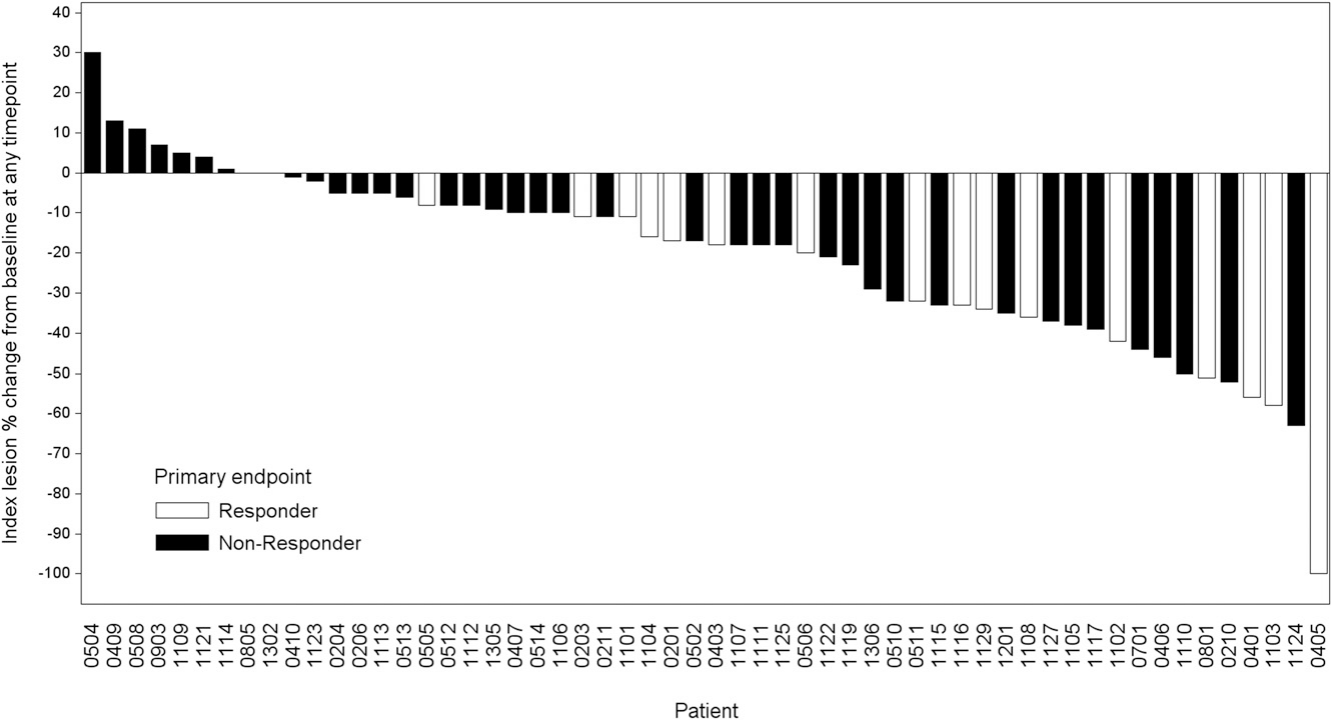
Maximum reductions in baseline tumor lengths of 56 patients with advanced PPGL who had measurable target lesions.

Of the 56 patients who had measurable target lesions, all 16 patients (100%) who had a reduction in baseline antihypertensive medication also had PRs or stable disease (Fig. 2). Among the 40 patients who did not achieve the primary endpoint and had measurable target lesions, 36 (90%) had PRs or stable disease, 3 (7.5%) had progressive disease, and 1 (2.5%) had unevaluable disease (Fig. 2).

### Biochemical Tumor Marker Response

Most patients (85%) who received at least 1 therapeutic dose of HSA ^131^I-MIBG had norepinephrine-secreting tumors. The proportions of patients who had biochemical tumor marker responses (CR + PR) increased over time, with the highest proportions observed 12 mo after the first therapeutic dose. At that time, for urine and serum norepinephrine, the overall response rates were 42% (8/19) and 31% (9/29), respectively, and the median response durations were 3.5 and 6.9 mo, respectively. For urine and serum normetanephrine, the overall response rates were 36% (9/25) and 44% (11/25), respectively, and the median response durations were 5.4 and 10.2 mo, respectively. For serum chromogranin A, the overall response rate was 68% (19/28), and the median response duration was 7.8 mo.

### OS

**FIGURE 3.**
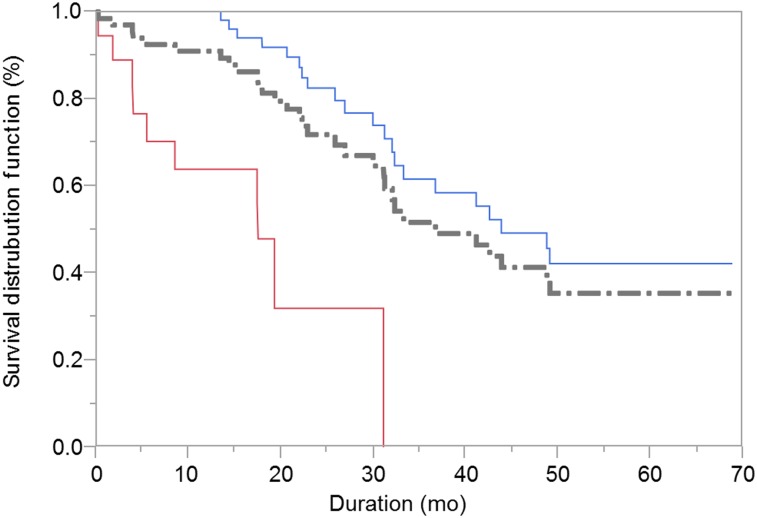
OS of 68 patients with advanced PPGL by number of therapeutic doses of HSA ^131^I-MIBG.

As of December 4, 2017, the median OS duration was 37 mo (95% CI, 31–49 mo). The Kaplan–Meier estimates of the 1-, 2-, 3-, and 5-y OS rates were 91%, 72%, 52%, and 36%, respectively (Fig. 3). The Kaplan–Meier curve appears to plateau at around 49 mo. The median OS duration of patients who received 2 therapeutic doses of HSA ^131^I-MIBG (44 mo; 95% CI, 32–>60 mo) was longer than that of patients who received 1 dose (18 mo; 95% CI, 4–31 mo). The median survival of patients with lung or liver metastasis at study entry (43 mo; 95% CI, 26–>60 mo) was similar to that of patients without lung or liver metastasis at study entry (41 mo; 95% CI, 31–>60 mo). The most common reason patients did not receive the second therapeutic dose was myelosuppression that did not subside over a period of 24 wk (according to protocol-specified criteria).

### AEs

Of the 68 patients who received at least 1 therapeutic dose of HSA ^131^I-MIBG, 67 (98.5%) had at least 1 treatment-related AE, including 20 patients (29%) who experienced at least 1 treatment-related SAE. The most common AEs were hematologic toxicity, nausea, vomiting, fatigue, dry mouth, dizziness, and headache (Table 3). Sixty-one patients (90%) experienced hematologic AEs, which were grade 3 or 4 AEs or SAEs in 49 (72%) of these patients. Although common, the hematologic toxicities resolved without the need for hematopoietic stem cell transplantation, which is typically indicated for prolonged myelosuppression. Only 17 patients (25%) required hematologic supportive care, which included packed red blood cell transfusions, platelet transfusions, granulocyte colony-stimulating factor, or erythropoietin therapy for a limited time.

**TABLE 3:** AE Incidence Among 68 Patients with Unresectable Advanced PPGL Who Received Any Therapeutic Dose of HSA ^131^I-MIBG

AE by preferred term	Treatment-related AE, all grades	Treatment-related AE, grades 3–5	Any AE, all grades
Nausea	52 (76)	1 (1)	53 (78)
Thrombocytopenia	49 (72)	28 (41)	49 (72)
Anemia	40 (59)	14 (21)	43 (63)
Leukopenia	41 (60)	28 (41)	41 (60)
Fatigue	32 (47)	7 (10)	41 (60)
Neutropenia	39 (57)	26 (38)	39 (57)
Vomiting	33 (49)	1 (1)	36 (53)
Dry mouth	27 (40)	0	28 (41)
Dizziness	16 (24)	1 (1)	27 (40)
Headache	15 (22)	0	21 (31)
Hypotension	8 (12)	1 (1)	18 (26)
Decreased appetite	14 (21)	1 (1)	17 (25)
Diarrhea	11 (16)	2 (3)	16 (24)
Constipation	4 (6)	1 (1)	16 (24)

Data are numbers followed by percentages in parentheses.

Grade 1 = mild AE; grade 2 = moderate AE; grade 3 = severe AE; grade 4 = life-threatening or disabling AE; grade 5 = death related to AE.

The most common treatment-related SAEs were hematologic toxicities, which occurred in 13 patients (19%). Other common treatment-related SAEs included pulmonary embolism in 2 patients (3%) and myelodysplastic syndrome in 3 patients (4%). Secondary malignancies included acute myeloid leukemia and acute lymphocytic leukemia in 1 patient each (1%). The acute myeloid leukemia case and 1 case of myelodysplastic syndrome were related to late radiation toxicity and resulted in death during the long-term follow-up phase (at 18 and 12 mo after the therapeutic dose, respectively). No patients had severe acute hypertension or hypertensive crises during or immediately after drug administration. During the 12-mo efficacy phase, 3 patients (4%) died of SAEs (1 patient each with perforated bowel, sepsis, and metabolic acidosis), none of which were considered treatment-related.

## DISCUSSION

Our study is the largest multicenter, prospective trial to use rigorous and standardized assessment criteria to evaluate the safety and efficacy of any therapy in patients with advanced PPGL. Our findings show that HSA ^131^I-MIBG conferred sustained control of catecholamine-associated hypertension in 25% of patients and had persistent antitumor effects in 22% of patients with advanced PPGL. All patients who had a sustained reduction in antihypertensive medication also had PR or stable disease as their best objective tumor response, a finding suggesting that HSA ^131^I-MIBG has broad antitumor effects.

Several patients exhibited some degree of objective tumor regression (including PRs) despite the fact that they did not meet the primary endpoint. Most of these patients had a reduction in their respective antihypertensive doses; however, the dose reduction was less than 50% when compared with baseline. Furthermore, some patients had a significant improvement in BP control at the same or a modestly reduced antihypertensive dose. These observations both indicate that HSA ^131^I-MIBG has antitumor effects that lead to a lower catecholamine secretion; however, because patients with metastatic PPGL usually have a large tumor burden, many will still need to be maintained on chronic antihypertensive therapy. Nevertheless, the hypertension will likely be more manageable and the associated risk for cardiovascular events will likely decrease while obtaining a long-term antineoplastic benefit.

Patients’ biochemical tumor marker responses further corroborate the objective tumor response achieved with HSA ^131^I-MIBG and offer mechanistic support for the treatment’s ability to reduce hypertension and thus yield a sustained reduction in antihypertensive medication. We also assessed OS, up to 5 y after treatment. In our study, patients who received at least 1 therapeutic dose of HSA ^131^I-MIBG had a median OS duration of nearly 37 mo. Patients who had lung or liver metastasis at study entry, despite their poorer expected prognosis (4,7), experienced a survival similar to that of patients who did not have such metastasis. However, these OS data should be interpreted with caution, as this was a single-arm study with no prespecified stratification.

Previous studies have investigated the use of conventional ^131^I-MIBG in patients with advanced PPGL (10–12,28,29). However, most of those studies were retrospective, had heterogeneous baseline patient characteristics, and had variable treatment protocols, making comparisons of their findings with those of the present study difficult. In a recent metaanalysis, van Hulsteijn et al. reported variable tumor response rates in 243 patients who received conventional ^131^I-MIBG at different doses in different treatment regimens (11). Overall, patients had PRs (27%) or stable disease (52%) at any time, although most of the reported responses lacked rigorous evaluation using RECIST 1.0. These findings are similar to those of an older retrospective analysis of ^131^I-MIBG by Loh et al. (10). In another retrospective review, 47.4% of treatment-naïve patients with metastatic PPGL had stable disease at 1 y (30). In contrast, all patients in the present study had advanced disease, a greater proportion of these patients (∼70%) had stable disease as defined by RECIST 1.0 at 1 y, and 30% of the patients who received 2 therapeutic doses had PRs during the 12-mo efficacy phase. Furthermore, among patients who received at least 1 therapeutic dose, the proportion of those who had PRs increased from 6% at 3 mo to 23% at 12 mo, indicating that HSA ^131^I-MIBG has persistent antitumor effects.

Overall, the AEs observed in the present study, including myelosuppression (including thrombocytopenia, leukopenia, and neutropenia) and gastrointestinal and constitutional symptoms of radiation exposure, were consistent with those observed in patients undergoing high-dose, conventional ^131^I-MIBG therapy. However, whereas up to 14% of PPGL patients receiving conventional ^131^I-MIBG therapy have severe acute hypertension during infusion (12), no patients in the present study had severe acute hypertension or hypertensive crises during or immediately after drug administration, likely because HSA ^131^I-MIBG contained a very small amount of unlabeled MIBG. Although the rate of hematologic AEs in the present study was somewhat higher than those reported previously, most of these AEs resolved without the need for further intervention. In addition, effects observed with HSA ^131^I-MIBG during the long-term follow-up phase, including myelodysplastic syndrome and secondary malignancies such as acute myeloid leukemia and acute lymphocytic leukemia, are also known to occur after cytotoxic therapies, including high-dose conventional ^131^I-MIBG and peptide receptor radionuclide therapy (12,31,32). Furthermore, many of the patients in this trial were heavily pretreated.

The present study was not without its limitations. First, the study did not include patients with advanced PPGL but no hypertension. This could have influenced the study’s findings or their interpretation and applicability by only allowing for the evaluation of a limited subset of the larger PPGL population. However, the ability to achieve objective tumor responses in patients who did not achieve the primary endpoint suggests that the etiology of hypertension in some patients with elevated BP may be unrelated to their oncologic disease. In addition, because this study was designed before the importance of mutations (particularly succinate dehydrogenase complex iron sulfur subunit B; *SDHB*) or hereditary syndromes was fully appreciated and samples were not routinely collected, we were not able to prospectively stratify by genetic background. Nonetheless, further analyses are being explored to fully characterize mutational status in any available patient samples. Accordingly, future studies could stratify and enrich the responder population by their mutational status (33). Also, given the challenges of identifying and recruiting patients with this rare disease over a period of many years, no effort could be made to control for the proportion of pheochromocytoma patients relative to paraganglioma patients, or to control for the imbalance in levels of a given biomarker that are not 1.5 times the upper limit of normal for a given assay or for the lack of clinical response. A more complete description of this secondary endpoint is beyond the scope of this article but will be included in subsequent publications as biomarker data are further analyzed. In addition, data are being analyzed to correlate patients’ prespecified characteristics and to explore the possibility of stratifying according to baseline characteristics in addition to those that were previously prestratified.

## CONCLUSION

Despite the above limitations, the data remain robust given the unique size of this prospective study for such rare tumors. The findings of the present study demonstrate that HSA ^131^I-MIBG can provide a clinical benefit to patients with advanced PPGL, as evidenced by improved BP control and by durable tumor and biochemical tumor marker responses. Collectively, our findings provide substantial evidence of the efficacy and safety of HSA ^131^I-MIBG therapy in patients with MIBG-avid advanced PPGL. To better understand the efficacy of this unique therapy, additional studies on normotensive PPGL patients or PPGL patients with specific genetic backgrounds are warranted.

The results of this trial impact our current clinical practice in a positive manner. A substantial number of patients with advanced PPGL have MIBG-avid tumors. As demonstrated by our study, most patients treated with HSA ^131^I-MIBG achieved tumor size reduction or disease stabilization for a prolonged duration. Furthermore, HSA ^131^I-MIBG was associated with BP improvement and an acceptable safety profile. As such, HSA ^131^I-MIBG offers an effective therapy for patients with this orphan disease. Chemotherapy with cyclophosphamide, vincristine, and dacarbazine and conventional MIBG have been used as off-label therapies. Clinical experience indicates that these therapies are associated with limited responses (∼30%). In addition, chemotherapy can lead to substantial toxicity. The results presented here suggest that HSA ^131^I-MIBG could then be a more effective (and the only Food and Drug Administration–approved) therapy to treat patients with inoperable locally advanced or metastatic PPGL. It is, however, important to recognize that a subset of patients with advanced PPGL may have tumors that are not MIBG-avid; in addition, some patients may not respond to MIBG therapy despite MIBG uptake. Therefore, scientific efforts must continue in the search for effective therapies for these patients.

## DISCLOSURE

Research funding was provided by Molecular Insight Pharmaceuticals, Inc., a wholly owned subsidiary of Progenics Pharmaceuticals, Inc. No other potential conflict of interest relevant to this article was reported.
