# Supplementary material for: Efficacy and Safety of High-Specific-Activity 131I-MIBG Therapy in Patients with Advanced Pheochromocytoma or Paraganglioma
Source: J Nucl Med. 2019 May;60(5):623–30. doi: 10.2967/jnumed.118.217463 (PMC6495236; doi:10.2967/jnumed.118.217463)

Supplemental Table 1: Estimated absorbed radiation doses (mGy/MBq) in 75 patients with unresectable advanced pheochromocytoma or paraganglioma who received a treatment-planning dose of high-specific-activity I-131 meta-iodobenzylguanidine (~5.0 mCi; ~185 MBq).

Target organ	Mean,	Minimum,	Maximum,	Standard deviation
Salivary glands	1.499	0.486	7.957	1.134
LLI wall	1.184	0.093	2.770	0.356
Thyroid	0.779	0.071	11.000	1.409
Urinary bladder wall[1]	0.614	0.141	0.930	0.142
ULI wall	0.514	0.091	1.120	0.138
Liver	0.509	0.180	7.830	0.862
Kidneys	0.360	0.085	0.772	0.163
Spleen	0.343	0.091	4.470	0.495
Lungs	0.323	0.123	3.170	0.344
Heart wall	0.272	0.073	1.550	0.215
Small intestine	0.194	0.085	0.347	0.042
Osteogenic cells	0.151	0.085	0.369	0.044
Gallbladder wall	0.146	0.083	0.852	0.094
Ovaries	0.126	0.000	0.271	0.046
Pancreas	0.117	0.068	0.484	0.054
Adrenals	0.116	0.067	0.535	0.059
Uterus	0.112	0.000	0.247	0.041
Stomach wall	0.100	0.059	0.279	0.033
Thymus	0.083	0.049	0.212	0.027
Muscle	0.082	0.049	0.188	0.024
Red marrow	0.079	0.048	0.175	0.022
Breasts	0.070	0.040	0.189	0.024
Skin	0.063	0.036	0.153	0.018
Testes	0.061	0.000	0.183	0.036
Brain	0.057	0.022	0.213	0.028
Total body	0.107	0.064	0.414	0.045

LLI, lower large intestine; ULI, upper large intestine.

[1]The mean bladder voiding interval was 4.8 hours.

Note: Data for 75 patients are provided because one patient underwent treatment planning twice.

Supplemental Figure 1: Scintigraphic images of MIBG-avid tumors.

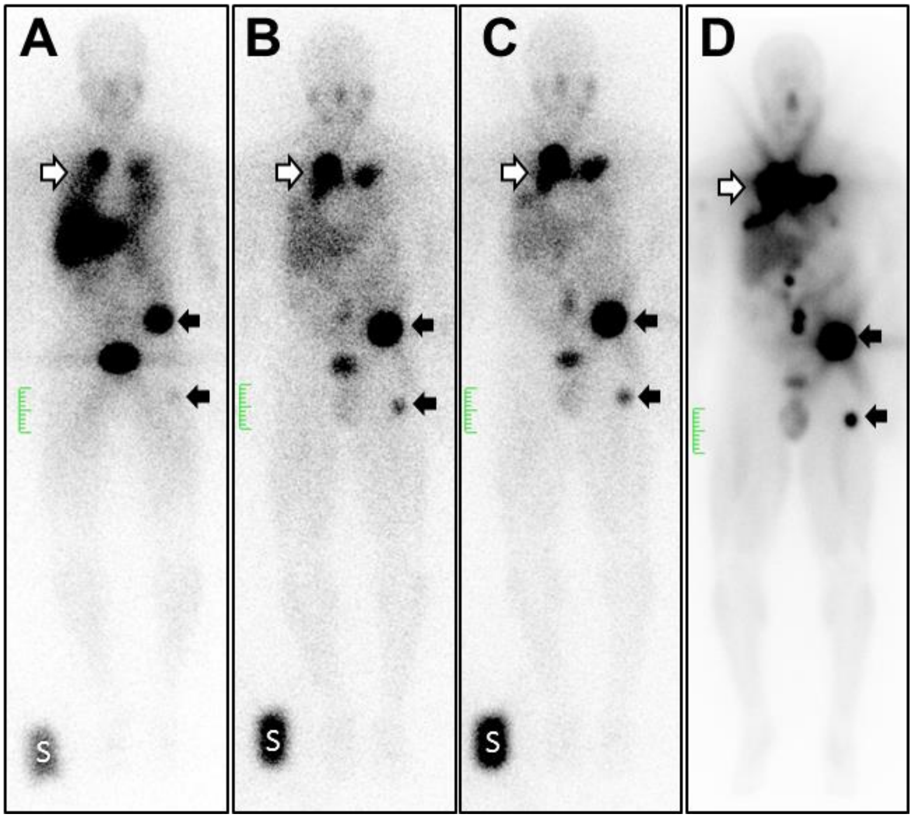

Panel 1:

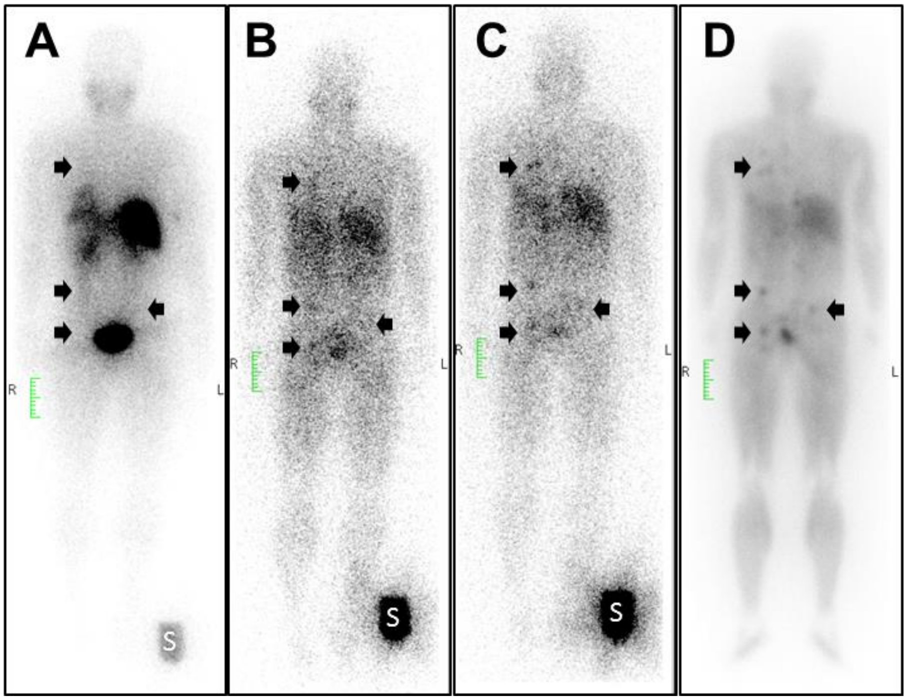

Panel 2:

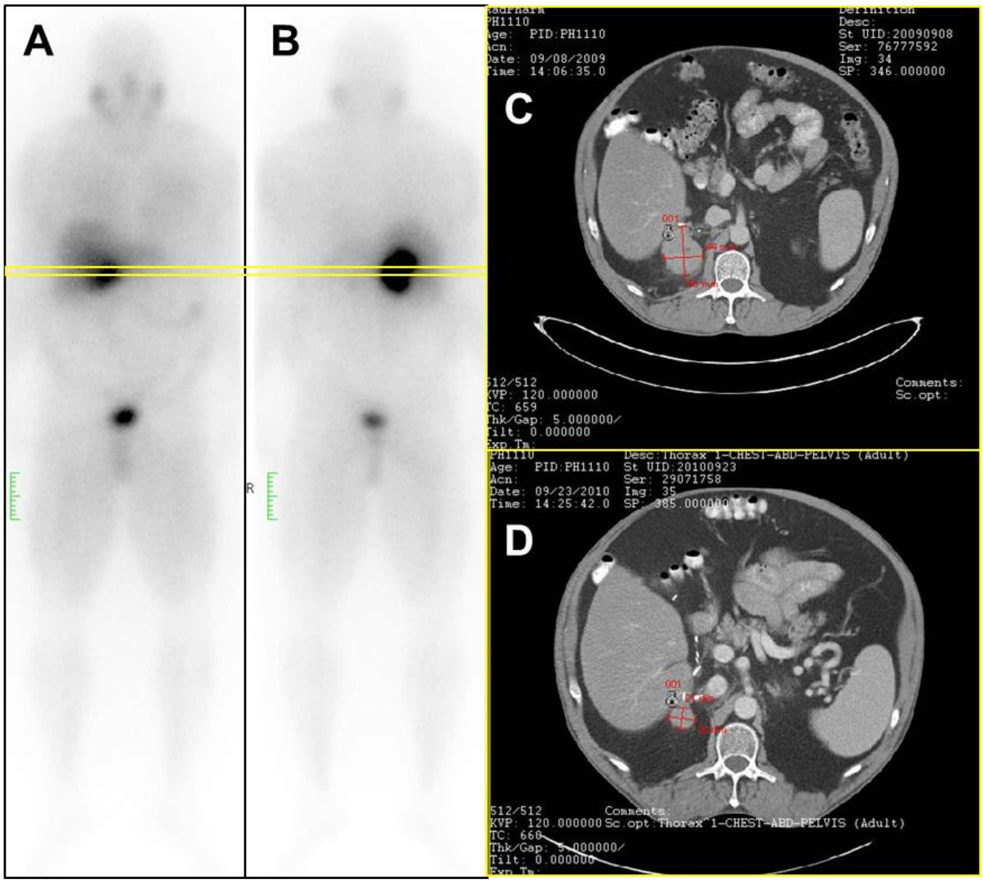

Panel 3:

Panel 1: Serial anterior whole body planar images of a 49 year old male with metastatic pheochromocytoma and imaging standard (S) for HSA I-131 MIBG therapy planning showing intense uptake in multiple skeletal (solid arrows) and soft tissue lesions (white arrow) at Day 1 (A), Day 2 (B), Day 3 (C), and a post-therapy anterior whole body planar image at 4 days following a therapeutic administration of 520 mCi (19,240 MBq) HSA I-131 MIBG (D).

Panel 2: Serial posterior whole body planar images of a 44 year old female with metastatic pheochromocytoma and imaging standard (S) for HSA I-131 MIBG therapy planning showing mild uptake in several bone metastases (solid arrows) at Day 1 (A), Day 3 (B), Day 4 (C), and a post-therapy posterior whole body planar image at 3 days following a therapeutic administration of 456 mCi (16,872 MBq) HSA I-131 MIBG (D).

Panel 3: Post-therapy anterior (A) and posterior (B) whole body planar images at 3 days following a therapeutic administration of 506 mCi (18,722 MBq) HSA I-131 MIBG in a 57 year old male with pheochromocytoma demonstrating intense uptake in a retroperitoneal soft tissue lesion. Corresponding slices on CT (yellow lines on the post-therapy scan) at baseline (C) and 12 months post-therapy (D) show that the longest diameter of the lesion decreased 50% from 5.6 cm to 2.8 cm. Patient's overall RECIST response was categorized as a PR.

Supplemental Figure 2: Duration of >50% reduction in antihypertensive use (full analysis set). The vertical line at 6 months indicates the duration needed to meet the primary endpoint.

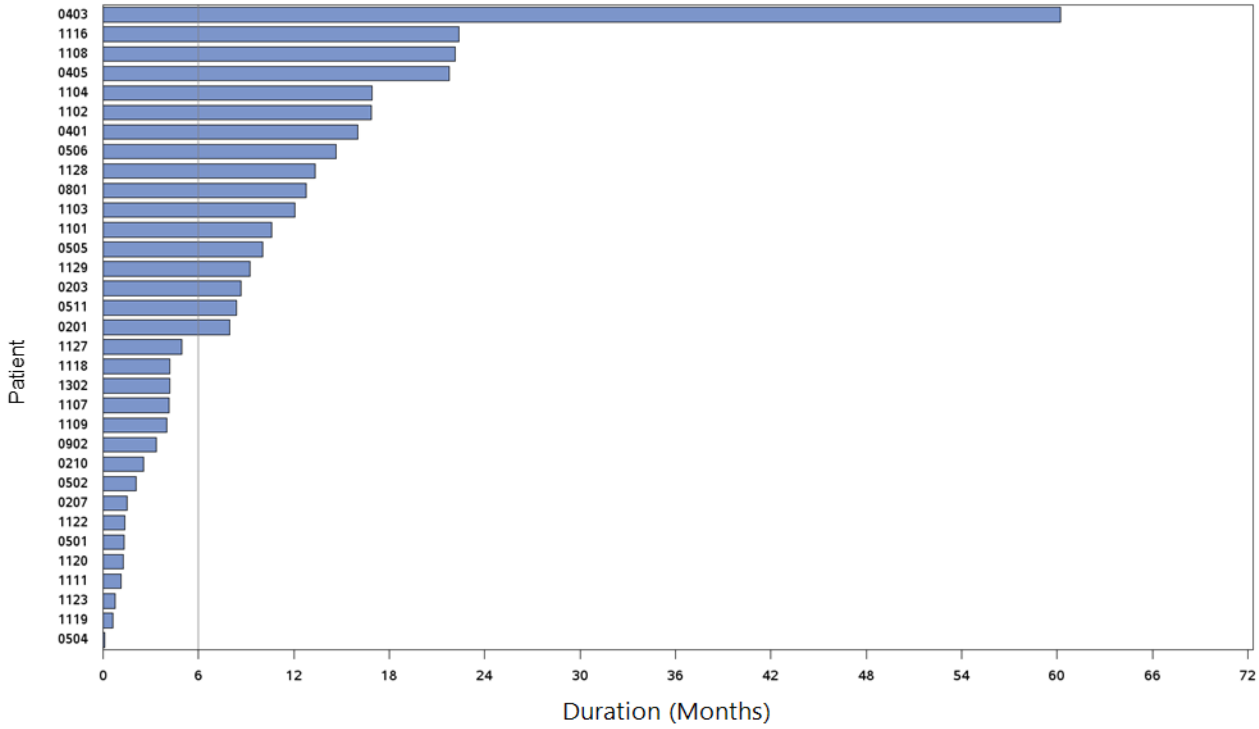

Supplement: Supplementary file 1 [file jnm217463SupplementalData.pdf]
